# Adoptive transfer of IL-4Rα^+ ^macrophages is sufficient to enhance eosinophilic inflammation in a mouse model of allergic lung inflammation

**DOI:** 10.1186/1471-2172-13-6

**Published:** 2012-01-31

**Authors:** Andrew Q Ford, Preeta Dasgupta, Irina Mikhailenko, Elizabeth MP Smith, Nancy Noben-Trauth, Achsah D Keegan

**Affiliations:** 1 Center for Vascular and Inflammatory Diseases, University of Maryland, Baltimore, 800 W. Baltimore St., Baltimore MD 21201, USA; 2 Marlene and Stewart Greenebaum Cancer Center and Dept of Microbiology & Immunology, University of Maryland School of Medicine, Baltimore MD 21201, USA; 3 Department of Biological Sciences, University of Maryland College Park, Rockville MD 20892, USA

## Abstract

**Background:**

The IL-4 receptor α (IL-4Rα) chain has a broad expression pattern and participates in IL-4 and IL-13 signaling, allowing it to influence several pathological components of allergic lung inflammation. We previously reported that IL-4Rα expression on both bone marrow-derived and non-bone marrow-derived cells contributed to the severity of allergic lung inflammation. There was a correlation between the number of macrophages expressing the IL-4Rα, CD11b, and IA^d^, and the degree of eosinophilia in ovalbumin challenged mice. The engagement of the IL-4Rα by IL-4 or IL-13 is able to stimulate the alternative activation of macrophages (AAM). The presence of AAM has been correlated with inflammatory responses to parasites and allergens. Therefore, we hypothesized that IL-4Rα^+ ^AAM play an active role in allergic lung inflammation. To directly determine the role of AAM in allergic lung inflammation, M-CSF-dependent macrophages (BMM) were prepared from the bone-marrow of IL-4Rα positive and negative mice and transferred to IL-4RαxRAG2^-/- ^mice. Wild type TH2 cells were provided exogenously.

**Results:**

Mice receiving IL-4Rα^+/+ ^BMM showed a marked increase in the recruitment of eosinophils to the lung after challenge with ovalbumin as compared to mice receiving IL-4Rα^-/- ^BMM. As expected, the eosinophilic inflammation was dependent on the presence of TH2 cells. Furthermore, we observed an increase in cells expressing F4/80 and Mac3, and the AAM marker YM1/2 in the lungs of mice receiving IL-4Rα^+/+ ^BMM. The BAL fluid from these mice contained elevated levels of eotaxin-1, RANTES, and CCL2.

**Conclusions:**

These results demonstrate that transfer of IL-4Rα + macrophages is *sufficient *to enhance TH2-driven, allergic inflammation. They further show that stimulation of macrophages through IL-4Rα leads to their alternative activation and *positive contribution *to the TH2-driven allergic inflammatory response in the lung. Since an increase in AAM and their products has been observed in patients with asthma exacerbations, these results suggest that AAM may be targeted to alleviate exacerbations.

## Background

Asthma is an inflammatory disease of the lung that has increased dramatically in western countries over the past 50 years [1-3]. Asthma is characterized by airway hyperresponsiveness (AHR), mucus hypersecretion, and airflow obstruction. Airways of asthmatics exhibit chronic inflammation with infiltration of the bronchial mucosa by lymphocytes, eosinophils, macrophages, and mast cells in conjunction with epithelial desquamation, goblet cell hyperplasia, and submucosal thickening. A number of genetically modified mouse strains have been used to understand the development of allergic inflammation in response to the model allergen ovalbumin [4-12]. T-cell deficient mice fail to develop lung inflammation and AHR, while B-cell deficient mice (and therefore IgE deficient) and mast cell deficient mice still develop these responses [5]. The importance of TH2 cells in the development of asthma in this model has been well established [13]. Treatment of mice with anti-IL-4, anti-IL-4Rα antibodies, or soluble IL-4Rα, inhibits the development of asthma [12,13]. As would be expected, IL-4Rα^-/- ^mice and STAT6^-/- ^mice also have greatly reduced asthma like symptoms in response to allergen challenge [5-11].

In the previous studies, TH2 differentiation was impaired explaining the lack of allergic inflammation. However, several groups showed that IL-4Rα^-/- ^mice have greatly reduced asthma like symptoms, even when purified TH2 cells were provided exogenously [7,8]. In addition, activating the receptor by transgenic expression or direct administration of IL-4 or IL-13 into the lungs in the absence of lymphocytes elicited symptoms of asthma including eosinophilia, mucus production, and AHR [7,14,15]. These results suggested that IL-4Rα on cells other than T cells are important in the development of asthma pathology. Using a bone-marrow chimera approach, we previously showed that the expression of the IL-4Rα chain on both bone marrow-derived and non-bone marrow-derived cells contributes to disease progression [16]. While expression of the IL-4Rα chain on either bone marrow- or non-bone marrow-derived cell types supported eosinophilic infiltration, expression of the IL-4Rα on both cell types resulted in the most severe inflammation [16]. Phenotypic analysis showed a strong correlation between the severity of allergic lung inflammation and the presence of IL-4Rα^+ ^macrophages [16].

In the presence of IL-4 or IL-13, IL-4Rα^+ ^macrophages adopt an alternative phenotype; such macrophages are termed alternatively-activated macrophages (AAM) [17-28]. These cells do not express potent pro-inflammatory mediators like the LPS/IFNγ treated classically activated macrophages (CAM). Rather, AAM express a different set of genes including *arg 1 *(arginase I), *Retnla *(Relma/FIZZ1), and the chitinase family members *Chi3L3/4 *(YM1/2), *Chi3L1 *(brp39) and produce the chemokines CCL2, CCL11, and CCL24 [20-29]. Furthermore, these AAM can fuse to form multinucleated giant cells (MNG) that act to sequester and degrade foreign materials [30,31]. These cells are found during TH2 responses in vivo, and are thought to be important for immune responses to helminthic parasites and for tissue repair [24-28,32].

Several in vivo studies established a correlation between the presence of AAM and inflammation. Mice infected with *Schistosoma mansoni *developed granulomatous liver pathology that correlated with high arginase activity [33]. Furthermore, *Heligmosomoides polygyrus *infection of mice elicited a TH2 response and AAM accumulated at the site of inflammation [32]. The presence of AAM also correlated with recruitment of eosinophils to the lung and peritoneal cavity in mice infected with *Nippostrongylus brasiliensis *(Nb) or with Sendai virus [34,35]. A reduction in macrophage numbers resulted in reduced eosinophilia in both models. Several strategies to delete the IL-4Rα simultaneously on monocytic and granulocytic cells also suggested a role for AAM in mediating eosinophilic inflammation in parasite infection models [36]. A study characterizing the amplification of AAM formation by IL-33 also established a correlation between AAM and eosinophilia in a mouse model of allergic lung inflammation [37]. In addition, increases in AAM in the lungs of female mice have been linked to the increased susceptibility of female mice to asthma as compared to male mice [38,39]. Furthermore, studies in children undergoing severe asthma exacerbations demonstrated an increase in AAM in the lung [40-42].

These previous studies demonstrated a correlation between the severity of TH2-driven inflammation and the presence of IL-4Rα^+ ^macrophages and AAM [16,32-42]. However, they did not determine whether AAM were sufficient to amplify the inflammatory response. Since human asthmatics have elevated numbers of AAM and have increased levels of chitinase proteins in their blood and lung lavage fluid [43-49], we hypothesized that AAM directly contribute to the severity of lung pathology. To directly test this hypothesis, we prepared MCSF-dependent macrophages from the bone-marrow (BMM) of IL-4Rα^+/+ ^or IL-4Rα^-/- ^mice and transferred them to IL-4RαxRAG2^-/- ^recipient mice. TH2 cells were provided exogenously. Strikingly, the adoptive transfer of IL-4Rα^+/+ ^BMM was sufficient to enhance TH2-driven allergen-induced lung inflammation. These macrophages were detected in the lung tissue and were YM1/2- and Mac3-positive indicating the macrophages were differentiating to AAM in the TH2 environment. These results demonstrate that IL-4/IL-13 responsive macrophages positively contribute to the TH2-driven allergic inflammatory response in the lung and may serve as targets to reduce allergen-induced exacerbations.

## Methods

### Reagents

Recombinant murine IL-4 (mIL-4), recombinant human IL-2 (hIL-2), recombinant human IFNγ (hIFNγ), and macrophage colony stimulating factor (MCSF) were obtained from R & D Systems (Minneapolis, MN). Rat anti-mouse IL-4Rα (CD124) M1 antibody was obtained from BD Pharmingen (Franklin Lakes, NJ). Grade V Chicken Egg Albumin (OVA) was obtained from Sigma-Aldrich (St. Louis, MO). Low-Tox^®^-M Rabbit Complement was obtained from Cedarlane Laboratories (Burlington, NC). Rat anti-mouse IL-12 (p40/p70) and rat anti-mouse IFNγ were purchased from BD Pharmingen (Franklin Lakes, NJ).

### Preparation of macrophages

BALB/c, BALB/c RAG2^-/-^, and BALB/c DO11.10 × RAG2^-/- ^were purchased from Taconic Farms (Hudson, NY). BALB/c IL-4Rα^-/- ^and BALB/c IL-4RαxRAG2^-/- ^mice were bred in the animal care facility at the University of Maryland, Baltimore (UMB) [50]. All experimental procedures were performed under the auspices of our approved animal protocol in accordance to the guidelines issued by the Institutional Animal Care and Use Committee at the University of Maryland, Baltimore.

Bone marrow was isolated from the hind limbs of mice and cultured overnight in RPMI-1640 supplemented with 10% FBS, 100 units/mL penicillin, 100 μg/mL streptomycin, 2 mM glutamine. Non-adherent cells were collected by centrifugation and cultured at 1.0 × 10^6 ^cells/mL in the presence of 20 ng/ml MCSF to generate bone-marrow derived, MCSF-dependent macrophages (BMM). After 3 days in culture, fresh media was added. After 6 days in culture, the adherent cells were isolated using a cell scraper and collected by centrifugation. These cells were routinely > 95% F4/80^+^/CD11b^+ ^macrophages. BMM were washed and transferred to BALB/c IL-4RαxRAG2^-/- ^mice by intraperitoneal (IP) injection at 5 × 10^6 ^cells per mouse. BMM were cultured in complete RPMI at 1 × 10^6 ^cells per ml for 48 h in the presence or absence of IL-4 (10 ng/ml). The cells were harvested using a non-enzymatic chelator (Cellgro, Mediatech, Inc. Manassas, VA) and analyzed by FACS.

### Western blot

Spleens were harvested from recipient mice six weeks after BMM transfer or from control BALB/c mice (n = 4). Single cell suspensions were prepared and pooled. The cells were then cultured in the presence or absence of IL-4 (10 ng/ml) for 30 min. Cells were lysed and immunoprecipitated with rabbit anti-mouse STAT6 (M-20, Santa Cruz). The precipitates were washed in lysis buffer and solubilized in SDS sample buffer. The samples were separated on 7.5% SDS-polyacrylamide gels before transfer to a polyvinylidene difluoride (PVDF) membrane. The membranes were then probed with a monoclonal anti-phosphotyrosine antibody, RC20. The bound antibodies were detected using enhanced chemiluminescence (Amersham, Arlington, IL). The blots were stripped and re-probed with anti-STAT6.

### Preparation of TH2 cells

TH2-effectors were prepared in vitro as described [16,50]. Briefly, lymph nodes were isolated from BALB/c DO11.10 × RAG2^-/- ^mice and crushed into a single cell suspension. Spleens were isolated from BALB/c mice, crushed into a single cell suspension, and depleted of T-cells using anti-Thy 1.2 antibody (HO1349 supernatant) followed Low-Tox^®^-M Rabbit Complement. The T-depleted spleen cells were irradiated at 3000 RADs, and used as antigen presenting cells.

The lymph node cells (5.0 × 10^5^) were cultured with irradiated splenocytes at a 20-fold excess in the presence of 10 μg/mL anti-IL-12, 10 μg/mL anti-IFNγ, 50 ng/mL mIL-4, and 1 μM OVA peptide. The cells were stimulated for four days and passed at a 1:5 dilution in the presence of 20 U/mL hIL-2. After 4 days of rest, the lymph node cells were restimulated as described above with a 10 fold excess of splenocytes, followed by another 4 days of rest prior to intravenous (IV) transfer to recipient mice. These cells were routinely > 10% + for IL-4 production after restimulation [16].

### Asthma induction

TH2 cells were transferred to BALB/c IL-4RαxRAG2^-/- ^mice intravenously at 1.0 × 10^7 ^cells per mouse one day after the macrophage transfer. The subsequent day chicken egg ovalbumin (OVA) was adsorbed to aluminum hydroxide (Alum) at a 1:1 ratio by slow rotation for 30 min at room temperature and injected interperitoneally into the BALB/c IL-4RαxRAG2^-/- ^mice delivering 100 μg of OVA/Alum per mouse. The mice were boosted interperitoneally with 100 μg of OVA/Alum per mouse, prepared as previously described, 14 days after the initial priming [16]. The mice were exposed, to a 1.0% (w/v) OVA/PBS solution by nebulization for 20 min on days 19, 22, and 27.

### Evaluation of airway inflammation

Mice were anaesthetized 48 h after the last OVA challenge. Bronchial lavage was performed on each mouse by inserting a 1 mm tube into the trachea and flushing the lungs with 1 ml of PBS. The samples were centrifuged and the supernatant was used for cytokine analysis. The cellular component of the bronchoalveolar lavage (BAL) was resuspended in PBS. Total cell counts were determined. Cytospin preparations of the cells were stained with Diff-Quick (Dade Behring, Newark, DE) and differential cell counts were performed based on cell morphology and staining using light microscopic analysis.

### Lung histology, immunohistochemistry, and immunofluorescence

The lungs were prepared for histology by perfusing the right ventricle with PBS. The samples were fixed in 10% formalin for 2 h at room temperature and then stored in 70% ethanol. Paraffin sections of the samples were produced and stained with either hematoxylin and eosin (H&E) or periodic acid Schiff (PAS). The degree of lung inflammation was scored as previously described [16] ranging from 0 (no inflammation) to 3 (maximum pathology). Immunohistochemistry was performed as described [51] using rabbit anti-mouse YM1/2 polyclonal antibody (StemCell Technologies, Vancouver, BC), rabbit anti-mouse F4/80, rabbit anti-mouse Mac-3 (BD Pharmingen), hamster anti-mouse CD3ε monoclonal antibody-PE (BD Pharmingen), or control rabbit IgG (StemCell Technologies, Vancouver, BC).

Lung sections were also analyzed by immunofluorescence microscopy. The sections were de-paraffinized and treated with an antigen retrieval solution of 0.1 M citric acid, 0.1 M sodium citrate for 20 min. After changes in PBS, the slides were immersed in methanol/0.3% H_2_O_2 _for 30 min to exhaust endogeneous peroxidase activity. The sections were then preincubated with 10% Goat Serum for 20 min at room temperature, followed by Rat anti-Mouse Mac-3 (BD Pharmingen) or non-immune rat serum for 1 h at room temperature. After PBS washes, the secondary antibody Goat anti-Rat Alexa Fluor 555 (Molecular Probes by Life technologies) was applied for 30 min at room temperature. The slides were rinsed in PBS and incubated with rabbit anti-mouse YM1/2 (StemCell Technologies Inc.) or non-immune rabbit serum for 1 h at room temperature, followed by goat anti-Rabbit Alexa Fuor 488 (Invitrogen) for 30 min. The slides were rinsed in PBS and incubated with DAPI for 2 min. Fluorescently stained slides were viewed on a Nikon E-800 fluorescent microscope using a Nikon 40×/0.75 Plan Fluor objective. Blue (DAPI), red (Mac3), and green (YM1/2) images of the same field were captured by a Retiga EXi camera (Q imaging, Canada) operated by Volocity software (PerkinElmer Inc.) using identical capture settings for experimental and control slides. The 3 color images were merged. Five fields were analyzed for the total number of nuclei, total number of cells staining red only, number of cells staining green only, and number of cells with both colors (yellow). The percentage of cells in each category was calculated by dividing each number by the total number of nuclei per field.

### FACS analysis

To prepare single cell suspensions from whole lungs, tissues were subjected to DNAse (Roche) and collagenase (Worthington Biochemical) treatment for 30 min at 37°C as described previously [52]. Tissue digests then were passed through 40 μm cell strainers (BD Falcon). Cells were collected by centrifugation and washed three times in a PBS/2% FBS solution (FACS buffer). The cells were resuspended in FACS buffer and pushed through a filter cap. Fc receptors were blocked using a rat anti-mouse CD16/CD32 antibody (2.4 G2, BD Pharmingen, Franklin Lakes, NJ). Cells were then incubated for 30 min at 4°C in the presence of anti-F4/80, anti-CD11b, and anti-IL-4Rα. Cells were collected by centrifugation, washed three times and resuspended in FACS buffer. Flow cytometric analysis was performed using Becton Dickinson FACScan™ and Becton Dickinson FACSCalibur™ machines. Live cells were gated on forward by side scatter and analyzed for F4/80 by CD11b staining. The cells expressing *high *levels of both CD11b and F4/80 have been shown to be macrophages [52,53] and these cells were analyzed for expression of IL-4Rα. Data was analyzed using FlowJo software v8.5.3 (Treestar, CostaMesa, CA).

Single cell suspensions of BMM were incubated with Fc Block (2.4 G2, BD Biosciences) followed by staining with anti-Mac3-PE or isotype control. Cells left unstained or treated with isotype control antibodies were used as controls.

Antibodies used for flow cytometric analysis include: murine IL-4Rα-M1 (R-Phycoerythrin (PE) or FITC-conjugated rat anti-mouse CD124) (BD Pharmingen, Franklin Lakes, NJ), F4/80 (eBiosciences), rat anti-mouse CD11b (Integrin α_m _chain, Mac-1 α chain) monoclonal antibody-PE (BD Pharmingen), rat anti-mouse Mac-3-PE (BD), and rabbit anti-YM1 (StemCell Technologies, Inc).

## Results

### YM1^+ ^AAM are present in lungs of Chimeric mice receiving IL-4Rα^+ ^bone-marrow and TH2 cells

**Figure 1:**
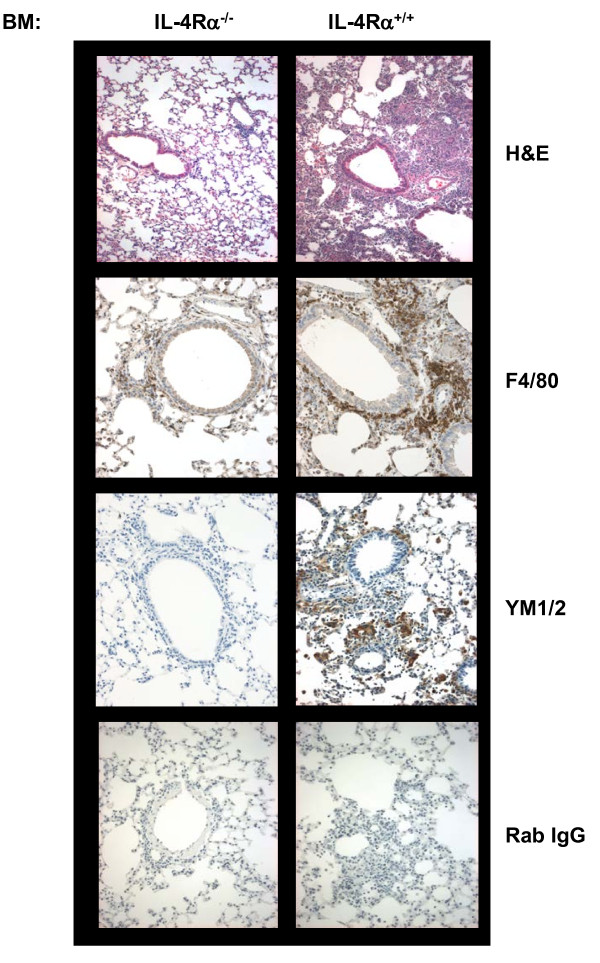
**AAM are present in the lungs of chimeric mice receiving IL-4Rα^+/+ ^bone-marrow**. Balb/c IL-4RαxRAG2^-/- ^mice were irradiated and reconstituted with bone-marrow cells isolated from IL-4Rα^-/- ^or IL-4Rα^+/+ ^mice as previously described [16]. Six weeks later, the mice were provided with OVA specific TH2 effectors (1 × 10^7^/mouse) by tail vein injection. They were then sensitized to 100 μg chicken egg ovalbumin (OVA) adsorbed to aluminum hydroxide (Alum) via i.p. injection on day 0 and boosted with the same reagent on day 14. Mice were exposed to 1% OVA in PBS by nebulization for 20 min each day on days 19, 22, and 27. Lung sections from OVA/alum primed mice were prepared and stained with H&E, anti-F4/80, anti-YM1/2, or control rabbit IgG as indicated. Digital images from a representative mouse (n = 4) for the 2 groups are shown (20X). These results are representative of 3 independent experiments.

Using bone-marrow chimeras, we previously demonstrated a strong correlation between the severity of allergic lung inflammation induced by the classic alum/ovalbumin prime/boost/challenge protocol and the presence of IL-4Rα^+ ^bone-marrow [16]. The enhanced inflammation was most evident when IL-4Rα^+/+ ^bone-marrow was transferred to IL-4Rα^-/- ^recipient mice in the presence of TH2 cells. Since bone-marrow derived macrophages adopt an alternative phenotype in response to the TH2 cytokines IL-4 and IL-13 [20], we analyzed lung sections from the OVA-primed chimeric mice for the expression of macrophage markers (Figure 1). We found that lung sections from IL-4RαxRag2^-/- ^mice receiving IL-4Rα^+/+ ^bone-marrow (BM) had greater inflammation and enhanced staining for F4/80 than lungs from mice receiving IL-4Rα^-/- ^BM. Furthermore, the lungs of OVA-primed mice receiving IL-4Rα^+/+ ^BM showed strong staining for the AAM marker YM1/2 while lungs from mice receiving IL-4Rα^-/- ^BM were negative for YM1/2. These results show that expression of IL-4Rα on bone-marrow derived cells is required for AAM formation during allergic lung inflammation in vivo.

### Transfer of IL-4Rα^+ ^BMM was sufficient to enhance TH2-driven allergic lung inflammation

**Figure 2:**
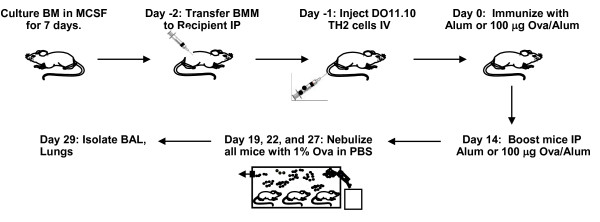
**Protocol for macrophage transfer and inflammation induction**. Bone-marrow derived macrophages (BMM) were derived from IL-4Rα^+/+ ^or IL-4Rα^-/- ^mice on the RAG2^-/- ^background as described in the Materials and Methods. BMM were washed and transferred to BALB/c IL-4RαxRAG2^-/- ^mice by IP injection at 5 × 10^6 ^cells per mouse. The next day, in vitro differentiated OVA-specific TH2 cells were injected IV at 1 × 10^7 ^per mouse. They were then sensitized to 100 μg chicken egg ovalbumin (OVA) adsorbed to aluminum hydroxide (Alum) via i.p. injection on day 0 and boosted with the same reagent on day 14. Mice were exposed to 1% OVA in PBS by nebulization for 20 min each day on days 19, 22, and 27.

Human asthmatics have elevated numbers of macrophages in their lungs and increased amounts of chitinase family members in their blood and lung lavage fluid [41-49]. An increase in AAM was observed in children undergoing severe asthma exacerbation [40]. Furthermore, the presence of AAM has been correlated with eosinophilic inflammation induced by infection and by allergen challenge [32-39]. Therefore, we hypothesized that the IL-4Rα^+ ^macrophages, capable of becoming AAM in the presence of IL-4 and IL-13, actively promote allergic inflammation rather than simply acting as bystanders. To test the direct contribution of IL-4Rα+ macrophages on the severity of allergic lung inflammation, we developed a macrophage transfer approach (Figure 2). We chose this transfer approach as opposed to a macrophage depletion approach because most depletion strategies are not completely specific for macrophages and they cannot maintain the depleted state over the length of the asthma protocol [34,36,54].

**Figure 3:**
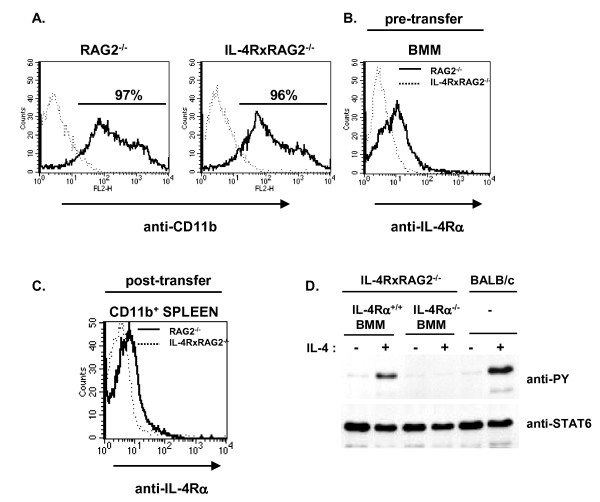
**Phenotype of macrophages pre- and post- transfer**. A. BMM were prepared from RAG2^-/- ^and IL-4RαxRAG2^-/- ^mice and were stained with anti-CD11b (solid line) or control antibody (dotted line) as indicated and analyzed by FACS. B. BMM were prepared from RAG2^-/- ^(solid line) and IL-4RαxRAG2^-/- ^(dotted line) mice and were stained with anti-IL-4Rα as indicated and analyzed by FACS. C. BMM were prepared from RAG2^-/- ^(solid line) and IL-4RαxRAG2^-/- ^(dotted line) mice and transferred to BALB/c IL-4RαxRAG2^-/- ^mice by IP injection at 5 × 10^6 ^cells per mouse. Six weeks later spleens were harvested and stained with anti-CD11b and anti-IL-4Rα and analyzed by FACS. Live spleen cells were gated on expression of CD11b; the IL-4Rα staining histograms are shown. These results are representative of 3 independent experiments. D. BMM were prepared from RAG2^-/- ^(IL-4Rα^+/+^) and IL-4RαxRAG2^-/- ^(IL-4Rα^-/-^) mice and transferred to IL-4RαxRAG2^-/- ^mice by IP injection at 5 x10^6 ^cells per mouse. Six weeks later spleens were harvested and single cell suspensions were prepared. In addition, single cell suspensions were prepared from untreated BALB/c mice. Pooled spleen cells were cultured in the presence or absence of IL-4 (10 ng/ml) for 30 min as indicated. Cell lysates were prepared and immunoprecipitated with anti-STAT6. The precipitates were analyzed by western blotting with anti-phosphotyrosine antibody. The membranes were stripped and re-probed with anti-STAT6.

We utilized bone-marrow-derived, M-CSF dependent macrophages (BMM) for transfer. We found that BMM prepared from either RAG2^-/- ^or IL-4RαxRAG2^-/- ^mice were routinely > 95% CD11b^+ ^(Figure 3A); as expected BMM isolated from RAG2^-/- ^mice expressed the IL-4Rα while those isolated from IL-4RαxRAG2^-/- ^mice did not (Figure 3B). IL-4Rα^+ ^BMM respond to IL-4 or IL-13 by inducing classic AAM genes while IL-4Rα^-/- ^BMM do not [20,55]. To determine whether transfer of BMM was feasible, we performed preliminary transfer studies. We found that IL-4Rα^+/+ ^BMM transferred into IL-4Rα^-/- ^mice by IP injection could be detected in the spleen up to 6 weeks after transfer (Figure 3C). Furthermore, splenocytes from mice receiving IL-4Rα^+/+ ^BMM, but not IL-4Rα^-/-^BMM, demonstrated the tyrosine phosphorylation of STAT6 in response to in vitro IL-4 treatment, albeit at reduced levels when compared to control BALB/c splenocytes (Figure 3D). These results demonstrate that macrophages adoptively transferred intraperitoneally are capable of populating lymphoid tissue.

**Figure 4:**
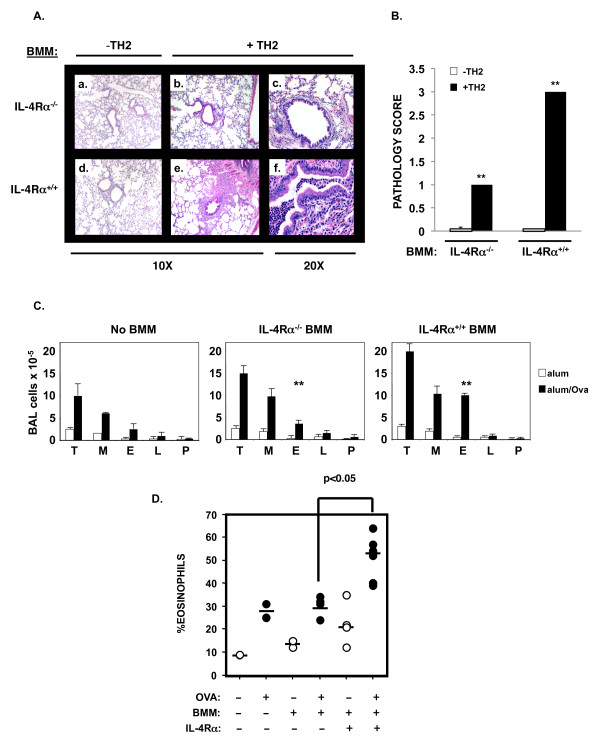
**Transfer of IL-4Rα^+/+ ^BMM enhances the TH2-driven, OVA-induced eosinophilic inflammation in the lungs and airways of mice**. BMM were prepared from IL-4Rα^+/+ ^or IL-4Rα^-/- ^mice as described above. BMM (5 × 10^6^) were transfered by IP injection to IL-4RαxRAG2^-/- ^mice on day -1. On day 0 mice were injected with PBS alone or with TH2 cells derived from D011.10 mice as indicated (1 × 10^7^). The mice were immunized with OVA/alum on day 1 as indicated, followed by boost and challenge as described in Figure 2. Lung sections from OVA/alum primed mice were prepared and stained with H&E. A. Several digital images (A-F) from a representative mouse from each group (n = 4) are shown at 10 and 20X. B. The slides from individual mice were evaluated for the degree of pathology (0-3) as described in Materials and Methods. The mean score for each group is represented in the form of bar graphs ± SEM. In most cases the SEM was too small to show on the graph. Significant differences between the two groups noted were determined using the Student's *T*-test. ** (*p *< 0.01) C. BMM were prepared as described above and transfered by IP injection to IL-4RαxRAG2^-/- ^mice on day -1. On day 0 all mice were injected with TH2 cells derived from D011.10 mice (1 × 10^7^). The mice were immunized with alum or OVA/alum on day 1 as indicated, followed by boost and challenge as described in Figure 2. The BAL from individual mice (n = 3-4) was evaluated by differential counting after cytospin. Total numbers of cells present in the BAL are represented in the form of bar graphs ± SEM (T, total cells; M, macrophages; E, eosinophils; L, lymphocytes; and P, polymorphonuclear leukocytes). ** Significant differences between the two groups noted were determined using the Student's *T*-test (*p *< 0.01). D. The percentage of eosinophils in the BAL isolated from the individual mice is shown with the mean of the values represented by the bar. Significant differences between groups were determined using the Student's *T*-test. * (*p *< 0.05) These results are representative of 3 independent experiments.

To examine the impact of these BMM on allergic inflammation in vivo, we transferred BMM prepared from IL-4Rα positive or negative mice to IL-4RαxRAG2^-/- ^recipient mice and provided in vitro primed wild type TH2 effectors one day later by IV injection (Figure 2). These recipient mice were then immunized intraperitoneally with alum alone or OVA/alum and challenged with OVA as shown (Figure 2). The adoptive transfer of IL-4Rα^+/+ ^BMM resulted in severe TH2-driven, OVA-induced allergic lung inflammation in IL-4RαxRAG2^-/- ^recipient mice as shown in representative H&E images (Figure 4A, panels e, f), while transfer of IL-4Rα^-/- ^BMM resulted in lower levels of lung inflammation (Figure 4A, panels b, c). Little to no inflammation was observed in the absence of TH2-effectors, as expected (Figure 4A, panels a and d; Figure 4B). The transfer of IL-4Rα^+/+ ^BMM greatly enhanced the amount of TH2-driven, OVA-induced inflammation around airways and blood vessels in the lungs (pathology score: 3+) as compared to transfer of IL-4Rα^-/- ^BMM (pathology score: 1+) (Figure 4B). Numerous airways were filled with eosinophils and mononuclear cells in the lungs of mice receiving IL-4Rα^+/+ ^BMM (Figure 4A, panel f). Since in both cases the recipient mice lacked IL-4Rα, the lung epithelial cells were negative for PAS staining, indicating they were not producing mucus (data not shown) [56,57].

The cellular composition in the bronchoalveolar lavage (BAL) of individual mice was evaluated by differential staining after cytospin. Representative images are shown in Additional file 1: Figure S1. Mice that received TH2 cells alone or TH2 cells plus IL-4Rα^-/- ^BMM showed a similar pattern in BAL cell numbers after priming and challenge with OVA (Figure 4C). For example, in the absence of tranferred macrophages there were 6.1 × 10^5 ^macrophages and 2.5 × 10^5 ^eosinophils recovered in the BAL while in the presence of IL-4Rα^-/- ^BMM this was modestly increased to 9.7 × 10^5 ^macrophages and 3.6 × 10^5 ^eosinophils. The total cell number recovered in the BAL was increased ~1.5 fold in the presence of IL-4Rα^-/- ^BMM. However, mice that received TH2 cells plus IL-4Rα^+/+ ^BMM showed an altered BAL cell composition. In the presence of IL-4Rα^+/+ ^BMM there was an increase in total BAL cells as compared to the other 2 groups and this increase was largely due to a significant increase in eosinophils. On average there were 10.4 × 10^5 ^macrophages, similar to the IL-4Rα^-/- ^BMM group, and 9.8 × 10^5 ^eosinophils representing a greater that 2-fold increase in the number of eosinophils.

A similar trend was apparent when the percentage of eosinophils in the BAL was evaluated (Figure 4D). Transfer of TH2 effectors alone resulted in some airway eosinophilia (10% eosinophils in the BAL) in response to inhaled OVA (Figure 4D). This level of eosinophilia was enhanced by immunization with OVA to 30%. The addition of IL-4Rα^-/- ^BMM did not alter the level of TH2-driven eosinophilia. However, transfer of IL-4Rα^+/+ ^BMM resulted in an increase in airway eosinophilia in response to inhaled OVA (20%); this was further increased by OVA immunization to 55%. Thus, transfer of IL-4Rα^+/+ ^macrophages significantly enhanced TH2-dependent, OVA-stimulated airway eosinophilia.

### IL-4Rα^+ ^BMM adopt the alternative phenotype in vivo

**Figure 5:**
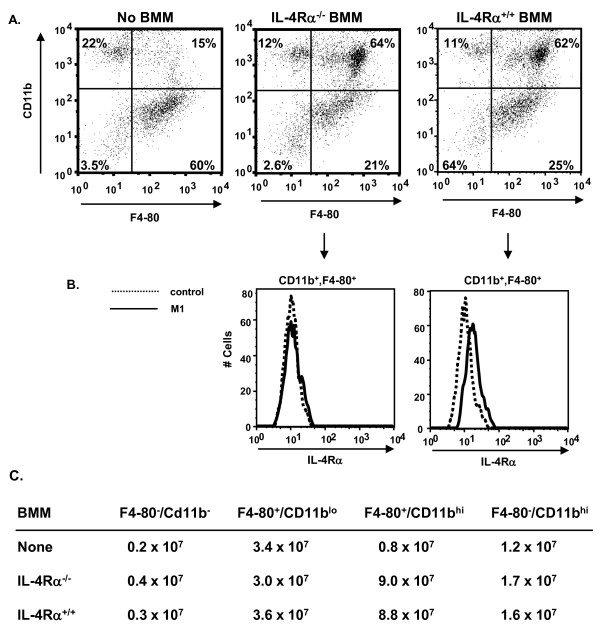
**Phenotype of macrophages in the lungs of recipient mice**. BMM were prepared from IL-4Rα^+/+ ^or IL-4Rα^-/- ^mice as described above. Where indicated BMM (5 × 10^6^) were transfered by IP injection to IL-4RαxRAG2^-/- ^mice on day -1. On day 0 all mice were injected with TH2 cells derived from D011.10 mice (1 × 10^7^). The mice were immunized with OVA/alum on day 1 as indicated, followed by boost and challenge as described in Figure 2. Lung digests were prepared from mice (n = 4) as described in Materials and Methods, pooled, and counted. Lung cells were stained with anti-CD11b, and anti-F4/80, along with anti-IL-4Rα or control IgG. A. Live cells were gated on forward by side scatter and analyzed for F4/80 by CD11b staining. B. The cells were gated for *high *expression of CD11b and F4/80 and analyzed for expression of IL-4Rα. The histograms comparing control staining (dotted line) to anti-IL-4Rα staining (solid line) of the gated cells is shown. C. The numbers of cells expressing the markers were calculated using the percentages of cells in each quadrant in Panel (A) and the total lung cells recovered. These results are representative of 2 independent experiments.

Lung digests were prepared from OVA immunized and challenged mice to analyze the recruitment of the transferred BMM to the lungs (Figure 5). Mice receiving either IL-4Rα^-/- ^or IL-4Rα^+/+ ^BMM showed an increase in the total numbers of lung cells recovered as compared to mice receiving TH2 cells alone from 5.6 × 10^7 ^to 14.0 × 10^7 ^and 14.2 × 10^7 ^total cells, respectively. Mice that received TH2 effectors alone showed few F4/80^+^, CD11b^hi ^cells (15%, 0.8 × 10^7^) in the lungs (Figure 5A, C). Our previous reports showed that F4/80^+^, CD11b^hi ^cells in the lungs are macrophages [52,53]. Mice receiving either IL-4Rα^-/- ^or IL-4Rα^+/+ ^BMM showed an increase in the percentages and the numbers of F4/80^+^, CD11b^hi ^cells in the lungs (64%, 9.0 × 10^7 ^and 62%, 8.8 × 10^7^, respectively). The F4/80^+^, CD11b^hi ^cells were positive for IL-4Rα expression in the mice that received IL-4Rα^+/+ ^BMM, but not in the mice that received IL-4Rα^-/- ^BMM (Figure 5B), showing that the transferred IL-4Rα^+/+ ^BMM were able to localize to the lung.

**Figure 6:**
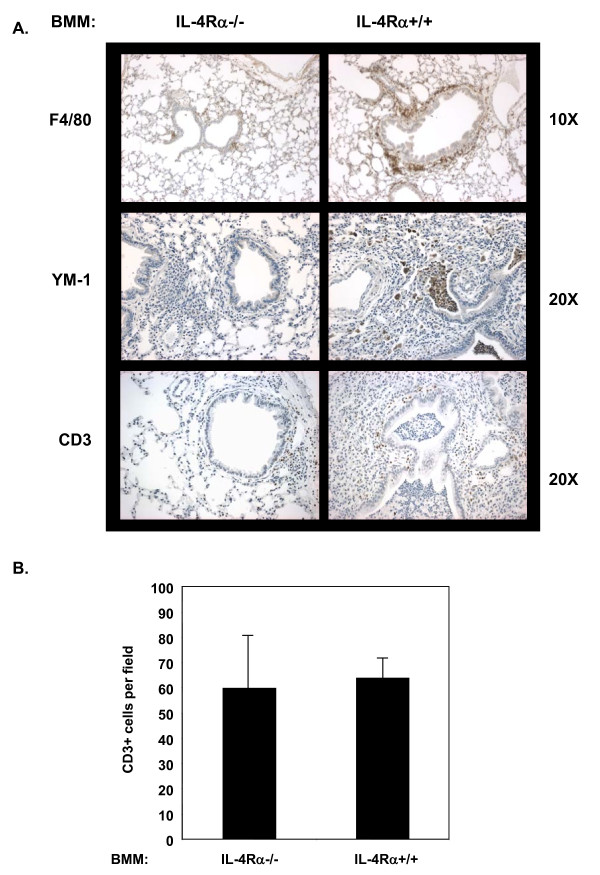
**Transfer of IL-4Rα^+/+ ^BMM leads to the presence of AAM in the lungs of mice**. A. Lung sections from the OVA/alum primed mice shown in Figure 4 were stained with anti-F4/80, anti-YM1/2, or anti-CD3 as indicated. Images from a representative mouse from each group are shown at 10X or 20X as indicated. CD3+ cells appear brown. B. The CD3+ cells in each 20X field were counted (n = 5). The average number of CD3+ cells per field were calculated and graphed. Data represented as cell counts ± SEM. These results are representative of 3 independent experiments.

To analyze the presence of AAM in the lung tissue, lung sections were analyzed by immunohistochemistry (Figure 6). The lungs of mice receiving IL-4Rα^+/+ ^BMM showed enhanced F4/80 staining of inflammatory cells surrounding airways and strong staining for YM1/2 in cells in the lung tissues and in the airways (Figure 6A). The YM1/2 staining was dependent on the presence of TH2 cells, since in their absence we did not detect any YM1/2 positive cells in the lungs of mice receiving IL-4Rα^+/+ ^BMM (Additional file 2: Figure S2). The lungs of mice receiving IL-4Rα^-/- ^BMM were largely negative for YM1/2 even though they showed modest inflammation. None of these sections were positive for iNOS, a marker of classically activated macrophages (data not shown). Taken together, these results show that the IL-4Rα^+/+ ^BMM were able to differentiate to the alternative phenotype in a TH2 environment. The lungs of mice receiving either the IL-4Rα^-/- ^or the IL-4Rα^+/+ ^BMM were equally positive for CD3 staining, indicating that the T-cells were recruited to the inflamed lung in both cases (Figure 6A, B).

**Figure 7:**
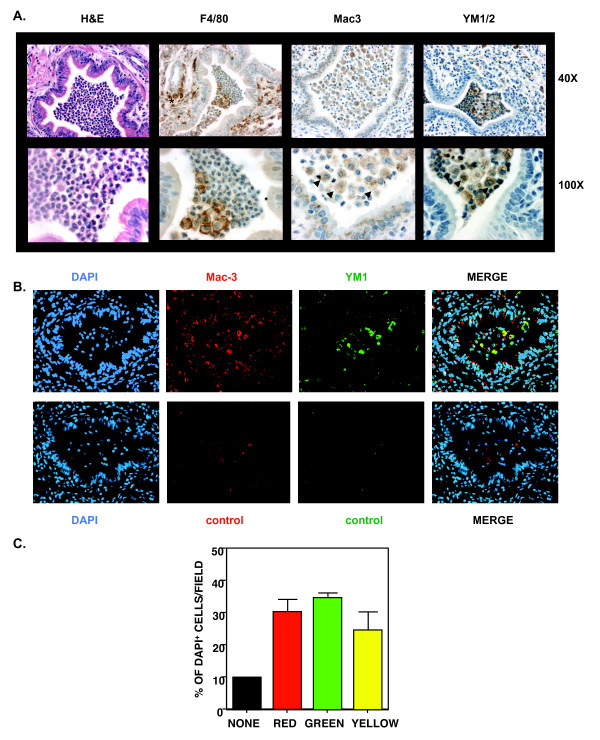
**YM1/2 and Mac3 staining coincide in the allergic lung**. A. Serial sections of lungs from a representative OVA/alum-primed mouse that received IL-4Rα^+/+ ^BMM were stained with H&E, anti-F4/80, anti-Mac3, or anti-YM1/2 as shown. Digital images were taken from the same region of the lung at 40X and 100X. Arrow heads highlight the staining of representative macrophages in the airways, while the asterisk highlights staining of macrophages surrounding blood vessels. B. Co-expression by immunofluorescence microscopy. A representative lung section was stained with rat anti-Mac3 and rabbit anti-YM1/2 (or control serum), followed by goat anti-Rat-PE and goat anti-rabbit-FITC. Nuclei were stained with DAPI. Digital images were captured for each color and processed as described in Materials and Methods. The 3 color images were merged into a single image. Appearance of yellow indicates expression of both Mac3 and YM1/2. C. Five fields were analyzed for the total number of nuclei, total number of cells staining red only, number of cells staining green only, and number of cells with both colors (yellow). The percentage of cells in each category was calculated by dividing each number by the total number of nuclei per field. These results are representative of 2 independent experiments.

To characterize the YM1/2^+ ^cells further, we performed immunhistochemistry on serial sections of the same lung region using antibodies against F4/80, Mac3, and YM1/2 (Figure 7A). Previous studies had shown that parasitic infection with *Nb *induced the expression of AAM genes in Mac3^+ ^macrophages in the lung suggesting that Mac3 (also known as CD107b and lysosomal-associated membrane protein 2, LAMP2) may also be induced by IL-4 and IL-13 [58]. Indeed, we found that IL-4 treatment of BMM in vitro increased expression of Mac3 (Additional file 3: Figure S3). Furthermore, both anti-Mac3 and anti-YM1/2 stained the large mononuclear cells found in close association with eosinophils in the airways and scattered in the lung tissue; several examples are marked by the arrow heads (Figure 7A). F4/80 also stained these cells, in addition to other cells marked by the asterisk surrounding the blood vessels; inflammatory cells surrounding blood vessels were largely negative for Mac3 and YM1/2. These results suggest that both Mac3 and YM1/2 can mark AAM in the inflamed lung, while F4/80 stains AAM as well as other inflammatory cells.

To determine whether macrophages co-expressed Mac3 and YM1/2, we stained the lung sections for both and analyzed them by immunofluoresence (Figure 7B). We found that a substantial fraction (25%) of the nucleated cells in the airway expressed both Mac3 and YM1/2. We also found cells expressing either Mac3 (30%) or YM1/2 (34%) alone and cells lacking expression (11%) suggesting heterogeneity in the macrophage population. These observations show that Mac3 and YM1/2 can be co-expressed in the inflamed lung.

### The presence of IL-4Rα^+/+ ^BMM enhanced the levels of TNFα and Chemokines in the BAL fluid

**Figure 8:**
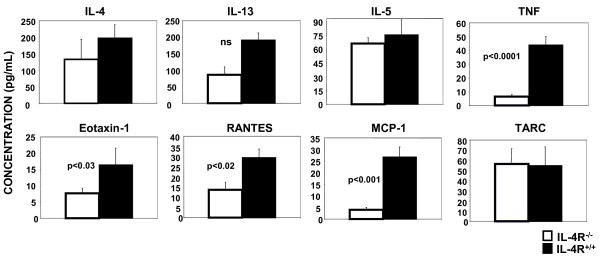
**Transfer of IL-4Rα^+ ^BMM alters cytokine and chemokine levels in the bronchalveolar lavage fluid (BAL)**. BMM were prepared from IL-4Rα^+/+ ^or IL-4Rα^-/- ^mice as described above. BMM (5 × 10^6^) were transfered by IP injection to IL-4RαxRAG2^-/- ^mice on day -1. On day 0 all mice were injected with TH2 cells derived from D011.10 mice (1 × 10^7^). The mice were immunized with OVA/alum on day 1 as indicated, followed by boost and challenge as described in Figure 2. The BAL fluid from individual mice (n = 3-4) was evaluated for cytokine and chemokine levels using Pierce Searchlight. The mean +/- SEM is shown. These results are representative of 3 independent experiments. Significant differences between groups was determined using the Student's *T*-test.

To determine whether the presence of IL-4Rα^+/+ ^BMM altered cytokine or chemokine production, the amount of cytokines and chemokines in the BAL fluid were analyzed using Pierce Search Light multiplex analysis (Figure 8). The concentrations of IL-4 and IL-5 in the BAL fluid were similar in mice receiving IL-4Rα^-/- ^or IL-4Rα^+/+ ^BMM, consistent with the presence of equal numbers of T-cells in the lung (Figure 6). There was a trend of more IL-13 in the BAL of mice receiving IL-4Rα^+/+ ^BMM, but this was not statistically significant. TNFα levels in the BAL fluid were greater in mice receiving IL-4Rα^+/+ ^BMM; the difference was statistically significant (*p *< 0.0001). Furthermore, the increased levels of eotaxin-1, RANTES, and MCP-1 in mice receiving IL-4Rα^+/+ ^BMM were statistically significant; levels of TARC were similar between the two groups.

## Discussion

The contribution of IL-4, IL-13 and IL-4Rα to allergic asthma disease progression and pathological characteristics has been examined in some detail [7,8,11,13]. However, the role of AAM in allergic lung inflammation has remained unclear. Several studies, including our own, have demonstrated a correlation between the presence of IL-4Rα+ macrophages and enhanced eosinophilic inflammation using infection and allergic models in mouse and man [16,32-47]. Previous studies have used clodronate loaded liposomes to deplete macrophages to demonstrate their role in infection-induced inflammation [35]. While informative, this approach is limited because clodronate can deplete any phagocytic cell, not only macrophages; the liposomes can induce a confounding inflammatory response on their own; and the depletion only lasts for 3 days [54].

In this study, we tested whether IL-4Rα^+ ^macrophages were sufficient to enhance allergen-induced eosinophilic inflammation. Since we provided OVA-specific, primed TH2-effectors, additional exposures to OVA would mimic allergen-induced exacerbation. Using this approach, we found that adoptive transfer of IL-4Rα^+/+ ^BMM, but not IL-4Rα^-/- ^BMM, significantly enhanced the TH2-dependent, OVA-induced recruitment of eosinophils to the lung. As would be expected in this model of inflammation, we found little to no inflammation in the absence of TH2 cells. Thus, in the presence of TH2 cells, transfer of IL-4Rα^+ ^macrophages is *sufficient *to enhance inflammation. However, it is not entirely clear whether they are the primary effectors of the enhanced inflammation or whether they stimulate another cell type.

We found that lungs from OVA-challenged IL-4Rα-deficient mice that received TH2 cells and IL-4Rα+ BMM showed positive staining for YM1/2. Immunohistochemistry of serial sections and immunofluorescence showed substantial overlap in staining of macrophages with YM1/2 and Mac3. The F4/80 staining showed a broader profile, overlapping with the YM1/2 and Mac3 staining, but also staining other inflammatory cells, especially cells cuffing blood vessels. Our results are consistent with those from the *Nb *infection model showing that Mac3+ cells express AAM markers [58]. However, whether Mac3 will be a reliable cell surface marker for AAM in inflamed tissues remains to be firmly established. Using immunofluorescence microscopy, we observed an interesting heterogeneity in expression of Mac3 and YM1/2 with Mac3^-^/YM1/2^-^, Mac3^+^/YM1/2^-^, Mac3^-^, YM1/2^+^, and Mac3^+^, YM1/2^+ ^cells present in the inflamed lungs. Whether these different subsets mediate different effector functions is unknown.

AAM have been shown to participate in other TH2 mediated inflammatory responses. Mice infected with nematode parasites induced the localized development of AAM in response to IL-4 [32]. Recruitment of eosinophils to the lung and peritoneal cavity in mice infected with *Nb *was dependent on macrophages; the absence of macrophages greatly prevented eosinophil recruitment [34]. Furthermore, intratracheal administration of in vitro differentiated AAM elevated the typically low level of allergic inflammation seen in male mice [39]. Taken together, our results and those from the other parasite infection models and allergic lung inflammation models strongly suggest that AAM are important, active contributors to inflammation and are not just bystander cells responding to the TH2 cytokines.

In our BMM transfer model, all host cells were IL-4Rα^-/-^, including resident lung macrophages, and thus were unable to respond to IL-4 or IL-13. We detected IL-4Rα+ macrophages in the lungs of mice that received IL-4Rα^+/+ ^BMM by intraperitoneal injection. In addition, we detected YM1/2+ macrophages in the lungs of these mice, but not in lungs of mice that received BMM lacking the IL-4Rα. These results show that the transferred BMM likely respond to the IL-4/IL-13 produced by the TH2 effectors in vivo and can localize to the lung. This is in contrast to studies using a parasite infection model reported by Jenkins et al. [59] where the majority of lung AAM was derived from proliferating resident lung cells and not from new immigrants. It is possible resident tissue macrophages proliferated in our model, however, since they lacked IL-4Rα, they would not be able to differentiate into AAM. The relative contribution of AAM derived from newly arrived inflammatory macrophages versus AAM derived from resident lung macrophages to eosinophilic inflammation remains to be established.

An important question arising from these studies is how the IL-4Rα^+ ^macrophages actively enhance the inflammatory response. IL-4/IL-13 stimulated macrophages make a number of factors including high levels of arginase 1, and chitinase and chitinase-like family members, however their contribution to allergic inflammation is not well understood [17-20]. Deletion of arginase 1 had no effect on the level of allergic inflammation [60]. Deletion of the chitinase family member brp39 profoundly suppressed allergic inflammation by an unknown mechanism [61]. Another chitinase like family member, YM1/2 has been shown to enhance TH2 cytokine production by T-cells [62].

IL-4/IL-13 stimulation of macrophages induces the production of eotaxin 1 and 2 (CCL11 and CCL24) that are important for the chemotaxis of eosinophils [29]. This chemokine production is amplified by IL-33 leading to enhanced eosinophilic inflammation [37]. AAM also produce MDC (macrophage-derived chemokine; CCL22) and TARC (thymus and activation-regulated chemokine; CCL17) that recruit macrophages and TH2 cells respectively [20]. IL-4 stimulation of AAM has been shown to enhance the production of MIP-1α (macrophage inflammatory protein-1; CCL3) a granulocyte chemokine [29]. The ability of AAM to produce chemokines may explain the enhanced recruitment of eosinophils we observed in mice receiving IL-4Rα^+/+ ^macrophages. We observed increased amounts of eotaxin-1, RANTES, and MCP-1 in the BAL fluid of mice receiving IL-4Rα^+/+ ^BMM, although the precise cellular source of these chemokines is not known. They could be derived from the AAM present in the lung and the BAL or they could be derived from another cell type responding to products made by the AAM.

In this regard, Medoff et al. found that an F4/80^- ^myeloid cell type was able to produce chemokines in a STAT6-dependent manner that enhanced *both *T-cell and eosinophil recruitment to the lung [63]. Since AAM have been shown to produce IL-13, it is possible the AAM are indirectly responsible for the increase in chemokines found in the BAL by producing IL-13 that can act on other myeloid cell populations in the lung [35]. Another group has shown that AAM regulate the activity of myeloid dendritic cells in female mice, suggesting a mechanism whereby females are more susceptible to lung inflammation than males [39]. While not a focus of this study, it is possible products made by AAM enhance the antigen presenting function of dendritic cells leading to enhanced T-cell activation. Clarification of the specific myeloid/macrophage population directly responsible for the enhanced eosinophilic inflammation observed after IL-4Rα^+ ^BMM transfer will require further investigation.

AAM may provide an environment hospitable to TH2 cells establishing a reciprocal relationship between these two cell types during the course of allergic inflammation. Such a relationship has been described as the innate-adaptive axis in asthma [35,41]. Chemokines produced by AAM such as TARC (CCL17) may enhance the recruitment of TH2 cells to the site of inflammation. Additionally, AAM increase expression of the IL-1 receptor antagonist (IL-1Ra), a decoy for IL-1 [17-20]. AAM can also produce IL-10 [17]. IL-10 suppresses IFNγ and STAT1 regulated responses. Since IFNγ can suppress TH2 differentiation, the IL-10 may act to perpetuate the Type 2 inflammatory response. Production of IL-1Ra and IL-13 by AAM may suppress the differentiation and function of TH17 cells [64]. In our model, where we provide high numbers of in vitro differentiated TH2 cells exogenously, these regulatory processes may not be apparent. We did not see a difference in the number of T-cells in the lungs or a difference in the amount of IL-4 or IL-5 in the BAL fluid between the IL-4Rα+ or IL-4Rα- transfer groups suggesting that the TH2 cells were being recruited and activated sufficiently in both settings. Additionally, there was no difference in the amount of IFNγ in the BAL fluid, while the levels of IL-17 were below detection in all groups (data not shown).

However, we did observe a significant increase in the levels of TNFα in the BAL of mice receiving IL-4Rα + BMM. While high levels of TNFα are typically associated with TH1-and TH17-mediated inflammatory diseases, TNFα levels have also been correlated with allergic inflammation in mice and with asthma severity in humans (reviewed in [65]. TNFα could play a role in the pathogenesis of asthma by stimulating a number of responses in inflammatory cells and structural cells including the recruitment and activation of eosinophils, the activation of mast cells, and the upregulation of adhesion molecules on endothelial and epithelial cells. The cellular source of our observed increase in TNFα in the BAL is unknown. Similar to the chemokines, many cell types are capable of producing TNFα including T-cells, macrophages, eosinophils, and epithelial cells [65]. AAM have also been shown to produce TNFα which can be enhanced by bacterial infection or LPS treatment [66,67]. Thus, it is possible the full set of cytokines and chemokines produced by AAM could favor TH2 dominance and promote a robust inflammatory environment in the lung.

## Conclusions

In summary, we have shown that transfer of IL-4Rα+ macrophages, but not IL-4Rα- macrophages, is sufficient to enhance TH2-driven allergic inflammation. Since AAM were only present in the lungs of mice receiving IL-4Rα+ BMM, these findings suggest that AAM contribute to the severity of allergic lung inflammation in the ovalbumin model. This contribution could be mediated by products made by AAM including chemokines and chitinase family members. An innate:adaptive axis has recently been appreciated in mice and humans, especially during acute asthma exacerbations [35,41]. It will be informative to delineate the most important mediators produced by AAM that lead to enhanced inflammation in mouse models and in humans.

## List of abbreviations

AAM: alternatively-activated macrophages; AHR: airway hyperresponsiveness; BAL: bronchoalveolar lavage; BMM: bone marrow derived macrophage; CAM: classically activated macrophages; CCL2: CC chemokine ligand 2; CCL11: CC chemokine ligand 11 (eotaxin-1); CCL24: CC chemokine ligand 24 (eotaxin-2); CCR3: CC chemokine receptor 3; FACS: fluorescence-activated cell sorting; γC: common gamma chain; IL-4: interleukin-4; IL-13: interleukin-13; IL-4Rα: interleukin-4 receptor alpha chain; iNOS: inducible nitric oxide synthase; IRS-2: insulin receptor substrate-2; LPS: lipopolysaccharide; MIP-1β: macrophage inflammatory protein-1beta; MNG: multinucleated giant cells; OVA: ovalbumin; PBS: phosphate-buffered saline; RT-PCR: RealTime PCR; PI-3K: phosphoinositide-3 kinase; RANTES: regulated on activation normal T-cell expressed and secreted; STAT6: signal transducer and activator of transcription 6; TARC: thymus- and activation-regulated chemokine; Tg: transgenic; TH2: T-helper 2; WT: wildtype.

## Authors' contributions

AQF and PD performed the experiments, analyzed the data, prepared the figures, and assisted with the preparation of the manuscript. EPS prepared the paraffin blocks and performed the immunohistochemistry and immunofluorescence staining. IM performed the immunofluorescence analysis, obtained the images, prepared the figure, and assisted with the preparation of the manuscript. NNT assisted in the design and execution of the experiments, and assisted with the preparation of the manuscript. ADK conceived and designed the study, assisted with the execution of the experiments, and wrote the manuscript. The authors have no competing interests to declare.

## Supplementary Material

Additional file 1 **Figure S1 Differential staining of cells in the BAL**. BMM were prepared from BALB/c RAG2^-/- ^(IL-4Rα^+/+^) or IL-4RαxRAG2^-/- ^(IL-4Rα^-/-^) mice by culturing in MCSF for 7 days. These BMM (5 × 10^6^) were transfered by IP injection to IL-4RαxRAG2^-/- ^mice on day -1. On day 0 all mice were injected with TH2 cells derived from D011.10 mice (1 × 10^7^). The mice were immunized with OVA/alum on day 1 as indicated, followed by boost and challenge as described in Figure 2. The cytospins of cells in the BAL were stained with Diff-Quick. Representative high power fields (100X) are shown. Click here for file

Additional file 2 **Figure S2 TH2-dependence of AAM differentiation in vivo**. BMM were prepared from IL-4Rα^+/+ ^mice and transferred (5 × 10^6^) by IP injection to IL-4RαxRAG2^-/- ^mice on day -1. On day 0 mice were injected with PBS alone or with TH2 cells derived from D011.10 mice as indicated (1 × 10^7^). The mice were immunized with OVA/alum on day 1 as indicated, followed by boost and challenge as described in Figure 2. Lung sections from OVA/alum primed mice were prepared and stained with anti-YM1/2. Images from a representative mouse from each group are shown at 40X or 100X as indicated (Arrows show YM1/2+ cells in the 100X image). Click here for file

Additional file 3 **Figure S3 Increased expression of Mac3 in IL-4-treated macrophages**. BMM were prepared from IL-4Rα^+/+ ^mice as described in Materials and Methods. The macrophages were cultured in the presence or absence of IL-4 (10 ng/ml) as indicated for 48 h. BMM were stained for Mac3 expression and analyzed by FACS. Click here for file
