# Supplementary figures and images for: Adoptive transfer of IL-4Rα+ macrophages is sufficient to enhance eosinophilic inflammation in a mouse model of allergic lung inflammation
Source: BMC Immunol. 2012 Jan 31;13:6. doi: 10.1186/1471-2172-13-6 (PMC3283450; doi:10.1186/1471-2172-13-6)

## Slide 1
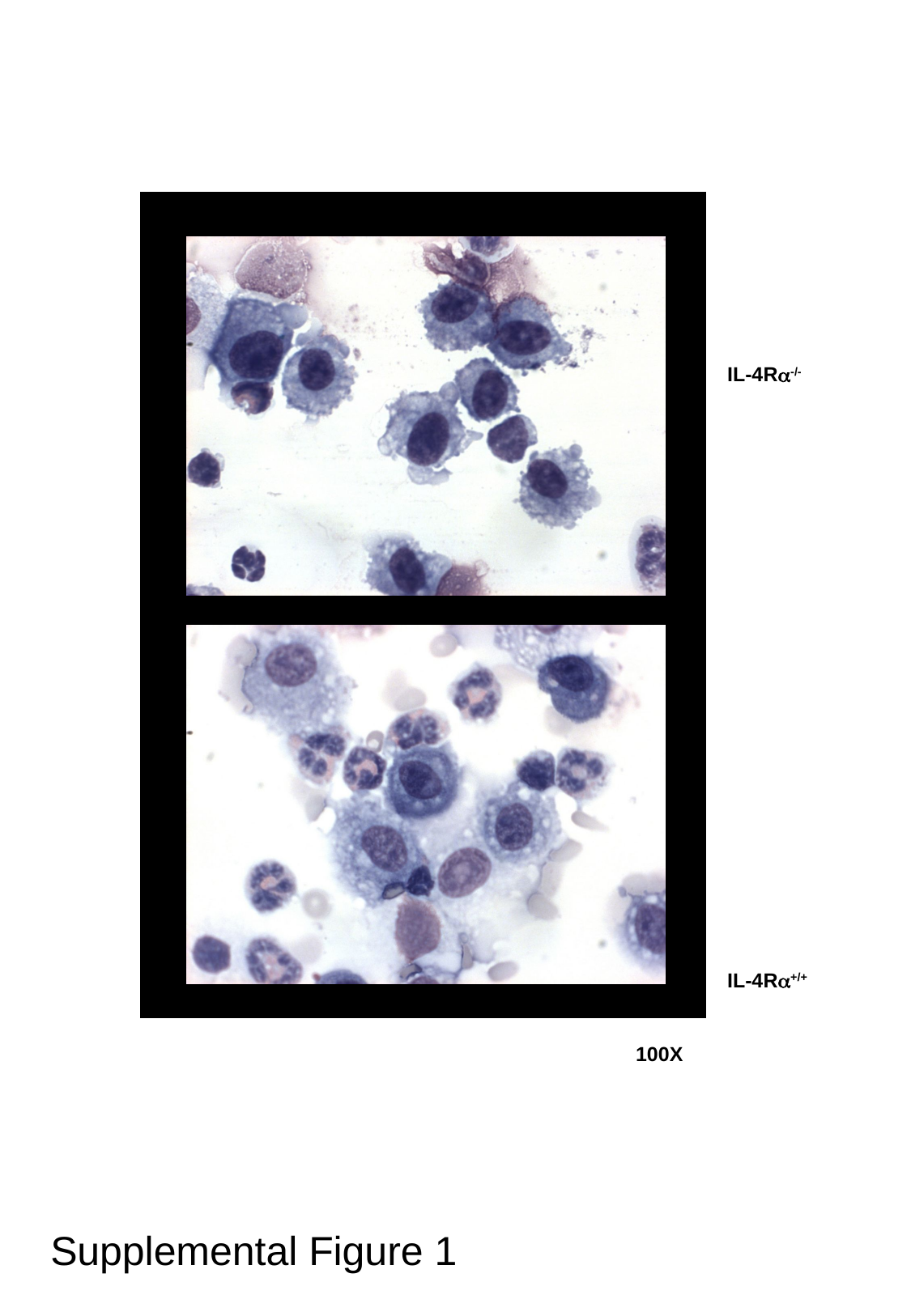

IL-4Rα-/-
IL-4Rα+/+
100X
Supplemental Figure 1

Supplement: Additional file 1 — Figure S1 Differential staining of cells in the BAL. BMM were prepared from BALB/c RAG2-/- (IL-4Rα+/+) or IL-4RαxRAG2-/- (IL-4Rα-/-) mice by culturing in MCSF for 7 days. These BMM (5 × 10^6^) were transfered by IP injection to IL-4RαxRAG2-/- mice on day -1. On day 0 all mice were injected with TH2 cells derived from D011.10 mice (1 × 10^7^). The mice were immunized with OVA/alum on day 1 as indicated, followed by boost and challenge as described in Figure 2. The cytospins of cells in the BAL were stained with Diff-Quick. Representative high power fields (100X) are shown. [file 1471-2172-13-6-S1.PPT]

## Slide 1
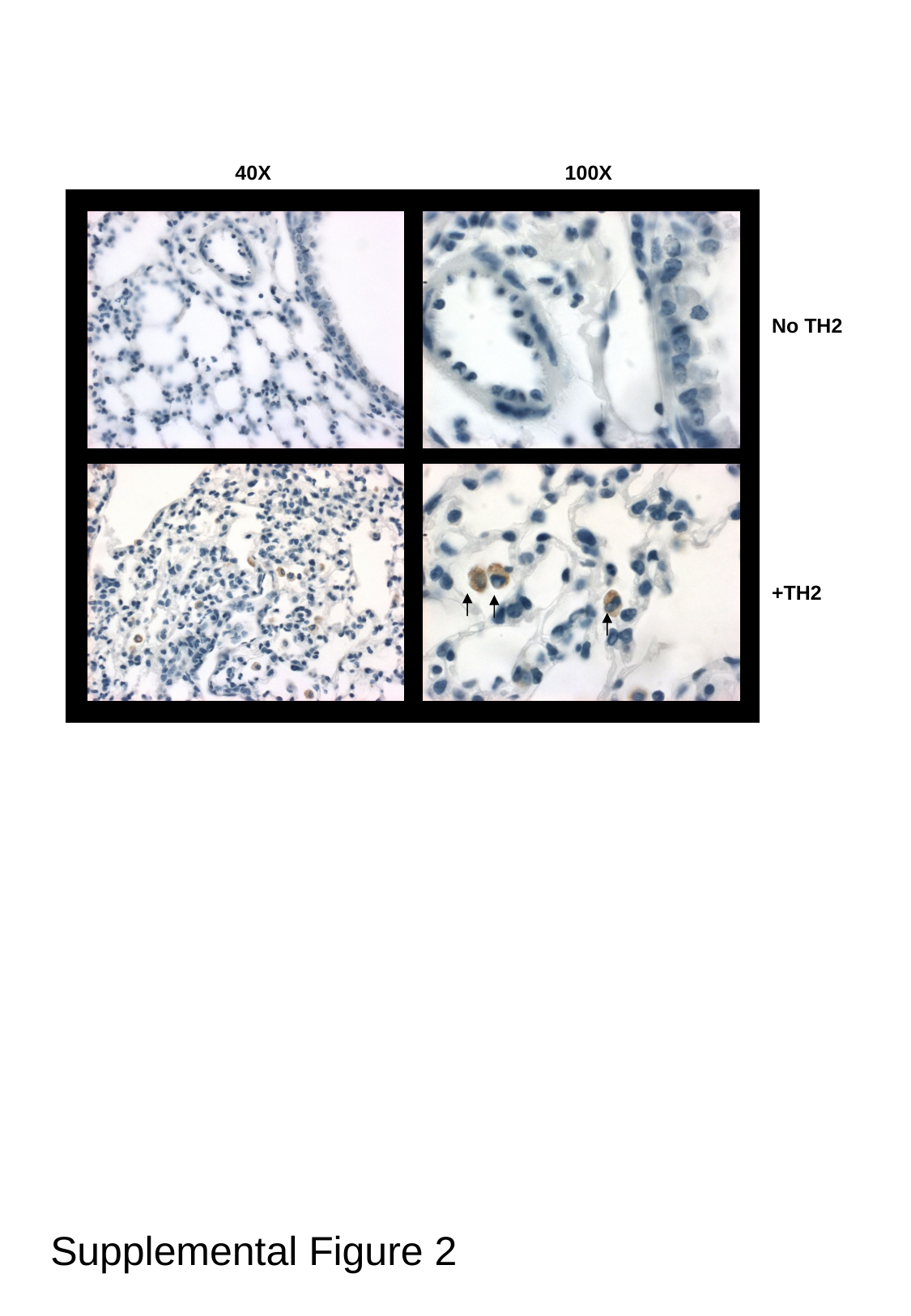

40X 100X
No TH2
+TH2
Supplemental Figure 2

Supplement: Additional file 2 — Figure S2 TH2-dependence of AAM differentiation in vivo. BMM were prepared from IL-4Rα+/+ mice and transferred (5 × 10^6^) by IP injection to IL-4RαxRAG2-/- mice on day -1. On day 0 mice were injected with PBS alone or with TH2 cells derived from D011.10 mice as indicated (1 × 10^7^). The mice were immunized with OVA/alum on day 1 as indicated, followed by boost and challenge as described in Figure 2. Lung sections from OVA/alum primed mice were prepared and stained with anti-YM1/2. Images from a representative mouse from each group are shown at 40X or 100X as indicated (Arrows show YM1/2+ cells in the 100X image). [file 1471-2172-13-6-S2.PPT]

## Slide 1
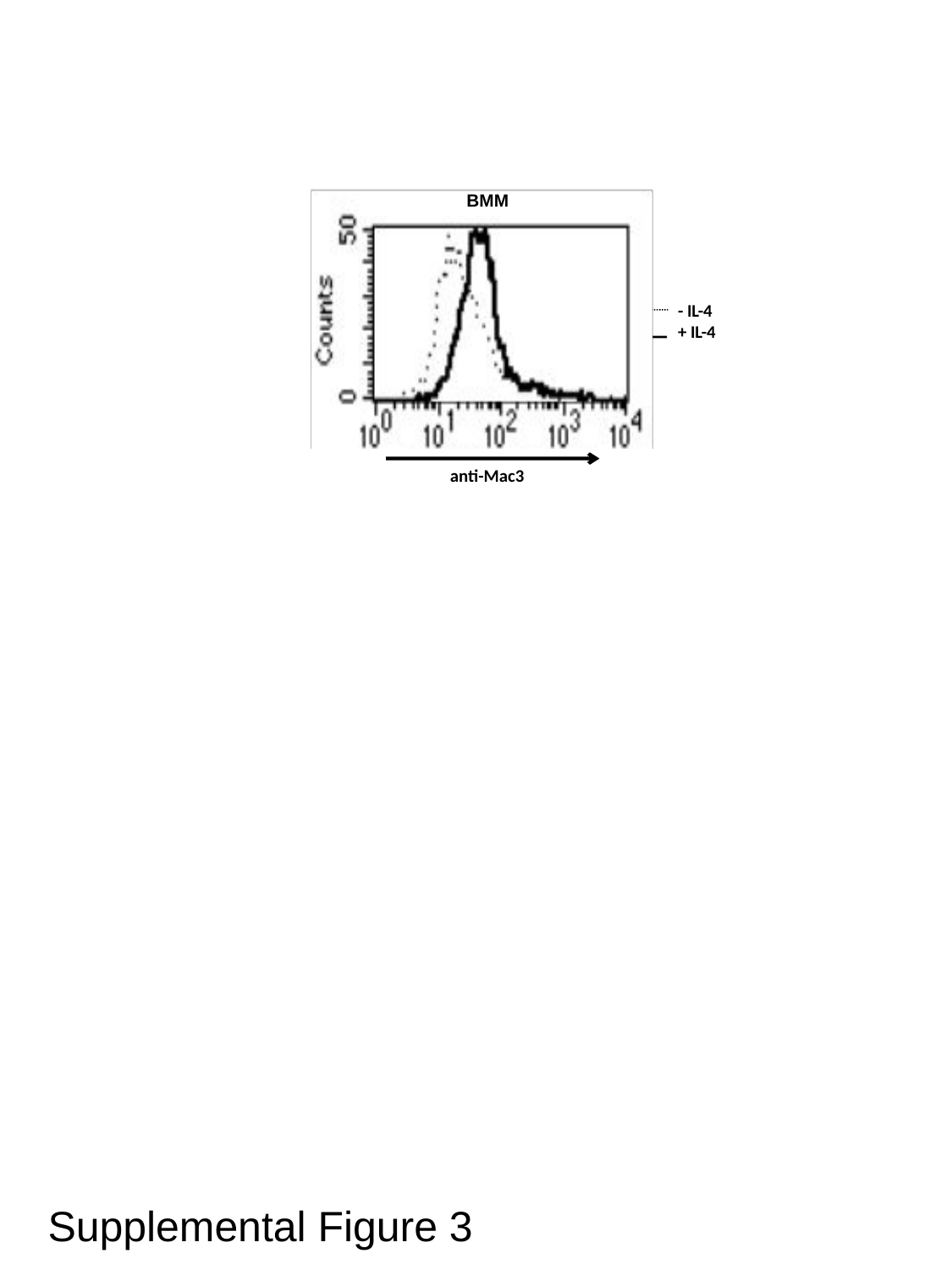

- IL-4
+ IL-4
anti-Mac3
BMM
Supplemental Figure 3

Supplement: Additional file 3 — Figure S3 Increased expression of Mac3 in IL-4-treated macrophages. BMM were prepared from IL-4Rα+/+ mice as described in Materials and Methods. The macrophages were cultured in the presence or absence of IL-4 (10 ng/ml) as indicated for 48 h. BMM were stained for Mac3 expression and analyzed by FACS. [file 1471-2172-13-6-S3.PPT]
